# Case of Obstinate Vomiting, Connected with the Presence of a Foreign Body in the Uterus

**Published:** 1866-10

**Authors:** James Blake

**Affiliations:** Professor of Obstetrics and Diseases of Women and Children in Toland Medical College, San Francisco


### SELECTIONS.

*Case of Obstinate Vomiting, connected with the Presence of a Foreign Body in the Uterus.* By JAMES BLAKE, M. D., Professor of Obstetrics and Diseases of Women and Children in Toland Medical College, San Francisco.

Dec. 18.—Was called to see Miss C., æt. 22, at 1 A. M. When I arrived, I found she had been suffering from constant vomiting for the last three days, for which she had been treated homœopathically, with the usual result; nothing was retained on the stomach, not even a drop of water or ice. There had been no sleep for the last two nights; in fact, the efforts to vomit never remitted for more than five minutes; nothing, however, was brought up except a little water or mucus. Expression of countenance haggard; tongue clean; skin cool; pulse 80, soft and weak; no pain except that caused by vomiting. I learned that the lady had been in ill health for several months, and that she had been treated for uterine disease. Ordered external application of turpentine over the pit of the stomach; chloroform, gtt. vj.; morph. sulph., gr. ¼; to be repeated every hour until the vomiting ceased.

10 A. M.—The first dose of the medicine quieted her, and she slept for a short time. A second dose was given at the end of the first hour, although there was no vomiting, and a third dose an hour afterward. This was rejected, and since then the vomiting has been as bad as ever. Ordered oxalate cerium, gr. iij., every hour. The mixture with chloroform and morphine to be repeated after four hours. As the bowels had not been moved for three days, I ordered an enema with castor oil and turpentine, to be followed, after its action, by one containing half a drachm of laudanum.

4½ P. M.—Much the same—patient getting weaker. As I was convinced that the vomiting was uterine, and the condition of the patient was such as to cause anxiety, I took an opportunity of inquiring as to the possibility of pregnancy existing, explaining my reasons for so doing. This was indignantly denied. I ordered brandy and soda water; enema with laudanum to be repeated.

19th.—Symptoms still the same; the laudanum enema had procured hardly any sleep; every thing is rejected a few minutes after it is swallowed; patient getting weaker, having retained no food or even water on the stomach for the last four days, during the whole of which time there has not been more than a few minutes' sleep. I learned to-day that the vomiting had come on after she had had something done to the womb, which caused her much pain, and that the surgeon had introduced the speculum after he had once withdrawn it, in order, as he stated, to remove a piece of cotton he had left in the womb. Thinking that the vomiting was kept up by the presence of a foreign body, I made a digital examination to see if I could discover it. I found the neck of the uterus enlarged and inflamed, the orifice patulous, and the body retroverted and flexed; could feel no foreign body. As it was time for menstruation to come on, I did not wash out the uterus as I otherwise should have done.

20th.—Rather better; had slept about an hour, and vomiting not so constant when lying quite still, but the slightest movement or attempt to speak, or opening of a door, or even being spoken to, brings on vomiting. Pulse 78, weak; skin cool; nothing retained on the stomach. I ordered champagne and brandy; carbonate of bismuth.

21st.—Better; slept some two hours during the night. Can take raw brandy, although water immediately vomits. States that when she feels the vomiting coming on, a tea-spoonful of raw brandy will check it. During the night there was a remission of the vomiting for three or four hours. Is better when lying on her right side; if she turns on her back, vomiting immediately comes on. Is now so weak that her voice is hardly audible. Ordered enemata of brandy and yolk of eggs. Menstruation came on last evening; had sharp, cutting pains at commencement, but was too weak to notice if any thing came away in the discharge, which is moderate.

For the next forty-eight hours the patient was kept alive by brandy and champagne, and nutritive enemata. It was not until the 24th that any food was retained on the stomach. At this time she was much prostrated, having been eight days without food, during the greater part of the time with constant vomiting and loss of sleep. The pulse was 85, small and weak; skin hot and dry—probably from the stimulants.

From this time the patient gradually recovered, although it was three weeks before she had gained sufficient strength to leave her room. Menstruation lasted the usual time, but was rather scanty. On making an examination per vaginam, about three weeks after menstruation had ceased, I found a piece of cotton in the vagina, such as is used for applying caustic to the interior of the cervix:

and I have no doubt but that this, whilst remaining in the uterus, had been the cause of the vomiting.

The above case affords a most striking example of the effect of mechanical irritation of the uterus in producing vomiting, and would tend to show that where pregnancy acts as a cause of vomiting, the vomiting is owing to the mechanical irritation produced by the fœtus, and not to the changes in the uterine system accompanying pregnancy.

The purely reflex nature of the vomiting in this case is interestingly shown by the causes that would give rise to it: the slightest movement, the opening of a door, even speaking to the patient, would bring on an act of vomiting, just as the same causes would give rise to spasm in tetanus or in poisoning by strychnine.—*Pacific* (*Cal.*) *Med. and Surg. Journal.*

